# Risk factors of re‐bleeding within a year in colonic diverticular bleeding patients

**DOI:** 10.1002/deo2.22

**Published:** 2021-08-25

**Authors:** Takahiro Gonai, Yosuke Toya, Keisuke Kawasaki, Shunichi Yanai, Risaburo Akasaka, Shotaro Nakamura, Takayuki Matsumoto

**Affiliations:** ^1^ Division of Gastroenterology, Department of Internal Medicine, School of Medicine Iwate Medical University Iwate Japan

**Keywords:** antithrombotic therapy, colonic diverticular bleeding, colonic diverticular hemorrhage, diverticular disease, lower GI bleeding

## Abstract

**Background/Aims:**

Although colonic diverticular bleeding (CDB) is common, few reports have described the effects of antithrombotic agents (ATs) on CDB. This study aimed to clarify the risk factors of re‐bleeding within a year in CDB patients.

**Methods:**

We retrospectively analyzed the risk of re‐bleeding in CDB patients. Among 324 patients who were hospitalized for acute lower gastrointestinal bleeding at our institution during the period from 2015 to 2019, we used 76 patients who were diagnosed as CDB. Risk factors for re‐bleeding were determined by Cox proportional hazard models.

**Results:**

Of 76 patients analyzed, 32 were taking ATs, nine of whom were taking multiple agents. Twenty‐six patients re‐bled within a year. Compared with the patients without re‐bleeding, patients with re‐bleeding within a year had been treated by antithrombotic therapy more frequently (62% vs. 32%, *p* = 0.013). Cox proportional hazard model revealed that treatment with ATs (hazard ratio 3.89, 95% confidence interval 1.53–10.74, *p* = 0.004) was an independent risk factor for re‐bleeding within a year.

**Conclusion:**

ATs were found to be an independent risk factor related to re‐bleeding within a year in patients with CDB.

## INTRODUCTION

Colonic diverticular bleeding (CDB) is common among patients with lower gastrointestinal bleeding.^1^ It has been presumed that the use of antithrombotic agents (ATs) and the use of non‐steroidal anti‐inflammatory drugs (NSAIDs) are risk factors for CDB, especially in the elderly population.^2^

In clinical practice, we often encounter patients with CDB in whom the responsible diverticulum cannot be identified due to the presence of numerous diverticula. On the other hand, a previous report revealed that spontaneous hemostasis occurred in 73% of patients with CDB,^3^ and their clinical course was uneventful without hemostatic treatment. However, in a certain proportion of patients with CDB, the hemodynamics become unstable due to continuous bleeding and re‐bleeding.

In patients with colonic diverticula, use of ATs including aspirin and NSAIDs has been shown to increase the risk of CDB.^4^, ^5^, ^6^, ^7^, ^8^ To date, however, only a few studies have investigated the effects of ATs on the re‐bleeding of CDB. In the present study, we aimed to clarify risk factors of re‐bleeding within a year in CDB patients.

## MATERIALS AND METHODS

### Patients

**FIGURE 1:**
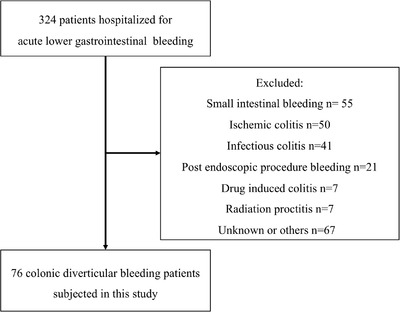
Flowchart of patients enrolled in this study. Three hundred twenty‐four patients were hospitalized for lower gastrointestinal bleeding. Two hundred forty‐eight patients were excluded for other diagnoses. Finally, 76 CDB patients were used in this study

This was a single‐center, retrospective study in which records of patients with CDB were reviewed. From January 2015 to August 2019, a total of 324 patients were hospitalized for acute lower gastrointestinal bleeding at our institution. Seventy‐six patients of them were diagnosed as CDB and enrolled in the present analysis (Figure 1). The primary outcome was re‐bleeding within a year.

The medical charts of the study patients were reviewed to collect their clinical and demographic characteristics, including age, sex, medical history, hospitalization, vital signs, endoscopic findings, transfusions, and medications. We further reviewed their subsequent clinical course at our institution until December 2019. Informed consent for study enrollment was obtained in the form of an opt‐out on the website. The study protocol was approved by the Institutional Review Board at Iwate Medical University (MH2019‐053).

### Diagnosis of CDB

We defined CDB as the presence of the following: (1) clinically obvious and visible melena with active bleeding from the colonic diverticulum confirmed with colonoscopy and (2) clinically obvious and visible melena without any colorectal bleeding lesion other than diverticula. We defined re‐bleeding as clinically obvious and visible melena occurring at least 24 h after cessation of initial bleeding, regardless of examination such as colonoscopy and computer tomography.

### ATs

ATs included antiplatelet and anticoagulant agents. Antiplatelet agents included aspirin, thienopyridine, eicosapentaenoic acid, and prostaglandin E1 derivatives. Anticoagulant agents included warfarin and direct oral anticoagulants (DOAC) such as edoxaban, apixaban, and riveroxaban.

The method of management of ATs for the study subjects depended on the decision of the attending physician. For the prevention of thromboembolic events, we replaced warfarin by heparin, and warfarin was restarted as soon as oral intake was permitted. Heparin was discontinued when the prothrombin time‐international normalized ratio was found to be 1.5 or higher after resuming warfarin.

### Statistical analysis

The clinical findings of the study patients were compared between two groups using the χ^2^ test, the Fisher exact probability test, and the Wilcoxon rank sum test. The re‐bleeding rate within a year was analyzed with the Kaplan‐Meier method, and differences between groups were assessed with the log‐rank test. Variables with a *p* value < 0.1 by univariate analysis were included in a Cox proportional hazard regression model. In each analysis, a *p* value < 0.05 was considered statistically significant. All statistical analyses were performed using JMP for Mac (Statistical Discovery Program, Cary, NC, USA).

## RESULTS

The clinical characteristics of the patients and the details of ATs are shown in Table 1. Forty‐six patients (60.5%) were men, and the median age of the patients was 75‐year‐old. The median duration of hospitalization was 8 days. Blood transfusion was requited for 29 patients. Endoscopic or other interventional hemostatic treatments were performed in 16 patients. Thirty‐two patients had been taking ATs, among whom nine patients had been taking two or more ATs. Eighteen patients discontinued ATs at the time of admission. Six of those patients had been taking warfarin; four of whom discontinued warfarin at admission. The period for warfarin discontinuance ranged from 3 to 9 days. Three of the four patients received heparin bridging. All of patients who discontinued ATs resumed ATs at the time of discharge. Thromboembolic event occurred in a patient during hospital stay after discontinuing ATs. The patient died of cerebral infarction.

**TABLE 1:** Clinical characteristics of patients and details of antithrombotic agents

Age, year, (median, range)	75 (38‐92)
Sex, male, *n* (%)	46 (60.5)
Hospitalization, days, (median, range)	8 (2‐43)
SAP, mm Hg, mean ± SD	129.4 ± 22.6
Minimum Hgb, g/dl, mean ± SD	9.4 ± 2.1
Blood transfusion, *n* (%)	29 (38.2)
Hypertension, *n* (%)	48 (63.1)
Diabetes mellitus, *n* (%)	17 (22.4)
Chronic kidney disease, *n* (%)	15 (19.7)
Malignant neoplasms, *n* (%)	5 (6.6)
Hemostatic treatment, *n* (%)	16 (21.1)
Re‐bleeding within a month, *n* (%)	13 (17.1)
Re‐bleeding within a year, *n* (%)	26 (34.2)
Antithrombotic agent, *n* (%)	32 (42.1)
Multiple antithrombotic agents, *n* (%)	9 (11.8)
NSAIDs, *n* (%)	13 (17.1)
Steroid, *n* (%)	8 (10.5)
Thromboembolic event, *n* (%)	1 (1.3)
Antithrombotic agents	
Antiplatelet agent, *n*	
Aspirin	11
Thienopyridine	16
Others	3
Anticoagulant agent, *n*	
Warfarin	6
DOAC	5

Abbreviations: DOAC, direct oral anticoagulants; Hgb, hemoglobin; NSAIDs, non‐steroidal anti‐inflammatory drugs; SAP, systolic arterial pressure; SD, standard deviation.

**FIGURE 2:**
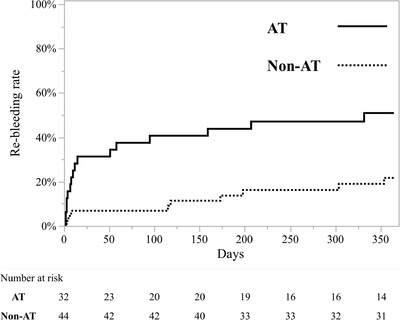
Re‐bleeding rate for patients taking antithrombotic therapy (AT; solid line) and not taking antithrombotic therapy (Non‐AT; dashed line) (*p* = 0.004)

Table 2 shows a comparison of clinical findings between patients with and without re‐bleeding within a year. The patients with re‐bleeding had been administered antithrombotic therapy more frequently than those without re‐bleeding (*p* = 0.013). Table 3 shows risk factors associated with re‐bleeding within a year. As shown in the table, use of ATs was found to be an independent risk factor of re‐bleeding (hazard ratio 3.89, 95% confidence interval 1.53–10.74, *p* = 0.004). Kaplan‐Meier curves showed that re‐bleeding within a year was significantly more frequent in the AT group than in the non‐AT group (50% vs. 23%, respectively; *p* = 0.004) (Figure 2).

**TABLE 2:** Clinical features of patients with or without re‐bleeding within a year

Variable	Re‐bleeding within a year (*n* = 26)	Without re‐bleeding (*n* = 50)	*p* value
Age, mean ± SD	73.8 ± 12.6	71.7 ± 12.5	0.37
Sex, Male, *n* (%)	16 (61.6)	30 (60.0)	0.90
SAP, mm Hg, mean ± SD	132.8 ± 26.1	127.7 ± 20.7	0.55
Hypertension, *n* (%)	17 (65.4)	31 (62.0)	0.77
Diabetes, *n* (%)	6 (23.1)	11 (22.0)	0.92
Chronic kidney disease, *n* (%)	7 (27.0)	8 (16.0)	0.26
Malignant neoplasms, *n* (%)	1 (3.9)	4 (8.0)	0.47
Hemostatic treatment, *n* (%)	4 (15.4)	12 (24.0)	0.37
Antithrombotic agent, *n* (%)	16 (61.5)	16 (32.0)	0.013
NSAIDs, *n* (%)	3 (11.5)	10 (20.0)	0.34
Steroid, *n* (%)	3 (11.5)	5 (10.0)	0.84

Abbreviations: NS, not significant; NSAIDs, non‐steroidal anti‐inflammatory drugs; SAP, systolic arterial pressure; SD, standard deviation.

**TABLE 3:** Cox proportional hazard model of risk factors for re‐bleeding within a year

Variable	HR	95% CI	*p* value
Antithrombotic therapy	3.89	1.53‐10.74	0.004

Adjusted factors: age, sex.

Abbreviations: CI, confidence interval; HR, hazard ratio.

## DISCUSSION

The results of our retrospective analysis indicated that ATs were associated with more frequent re‐bleeding within a year in patients with CDB.

It is known that ATs increase the risk of bleeding in patients with colonic diverticula.^4^, ^5^, ^6^, ^7^, ^8^ In contrast, it is known that discontinuation of aspirin and anticoagulants after the occurrence of gastrointestinal bleeding can increase the risk of cardiovascular events.^9^, ^10^, ^11^ Moreover, use of DOAC has been associated with gastrointestinal bleeding, with a risk equal to or even greater than warfarin.^12^, ^13^, ^14^ In patients with CDB, it has been reported that anticoagulants including DOAC are not associated with re‐bleeding, but their discontinuation increases the risk of ischemic stroke.^15^, ^16^, ^17^ However, a management strategy for ATs in patients with CDB has not been established.

The guideline for acute lower gastrointestinal bleeding proposed by the American College of Gastroenterology recommends that except for aspirin for primary prevention of cardiovascular events, antiplatelet agents should not be discontinued, and, in case of discontinuation, they should be resumed as soon as possible.^18^ The British Society of Gastroenterology recommends that warfarin and DOAC should be interrupted, except for patients at a high thrombotic risk.^19^ In the present study, approximately half of CDB patients under the use of ATs discontinued ATs at the time of admission, among whom a patient developed thromboembolic event.

This study evaluated risk factors associated with re‐bleeding within a year. A previous study reported that spontaneous hemostasis was seen in 73% of patients with CDB.^3^ In that study, 60 of 76 patients (73%) achieved spontaneous hemostasis without specific treatment. However, it has been reported that 20%–34% of patients with CDB experience re‐bleeding within a year.^3^, ^20^, ^21^, ^22^ Similarly, 34% of our patients experienced re‐bleeding within a year. We also showed that use of ATs was an independent risk factor for re‐bleeding in patients with CDB. In previous studies, several risk factors for re‐bleeding were identified in patients with CDB. Among those, the presence of signs of shock at initial bleeding was found to be a risk factor for re‐bleeding within 30–90 days.^23^, ^24^ In contrast, various risk factors have been reported to be associated with re‐bleeding 6 months or later.^20^, ^21^, ^22^ These reports suggest that risk factors for re‐bleeding are heterogeneous according to the time interval after onset. Our present study showed that CDB patients with ATs are at a higher risk of re‐bleeding within a year. It thus has been suggested that CDB patients with ATs should be observed more carefully during and after hospitalization. We thus should inform the attending doctor who prescribes ATs of a need for careful follow‐up after discharge.

The present study has several limitations. First, the retrospective design likely introduced selection bias. Second, the sample size was limited because this study was conducted at a single center. Due to the small sample size, we could not analyze the effects of continuation and discontinuation of ATs in CDB patients after CDB. A larger, multicenter cohort or prospective study is warranted to confirm our findings and clarify the effects of continuation/discontinuation of ATs.

## CONCLUSIONS

In conclusion, our study showed that ATs were found to be an independent risk factor related to re‐bleeding within a year in patients with CDB. Based on these findings, we suggest that patients with CDB receiving ATs should be observed more carefully during and after hospitalization.

## CONFLICT OF INTEREST

Authors declare no conflict of interests for this article.

## FUNDING INFORMATION

None.

## Data Availability

In this study, we did not get patients’ consent to release their data publicly, so supporting data is not available.

## References

[1] Niikura R , Nagata N , Shimbo T , *et al*. Natural history of bleeding risk in colonic diverticulosis patients: A long‐term colonoscopy‐based cohort study. Aliment Pharmacol Ther 2015; 41: 888–94. 25715746 10.1111/apt.13148

[2] Kinjo K , Matsui T , Hisabe T , *et al*. Increase in colonic diverticular hemorrhage and confounding factors. World J Gastrointest Pharmacol Ther 2016; 7: 440–6. 27602246 10.4292/wjgpt.v7.i3.440 PMC4986401

[3] Tanaka Y , Motomura Y , Akahoshi K , *et al*. Predictive factors for colonic diverticular rebleeding: A retrospective analysis of the clinical and colonoscopic features of 111 patients. Gut Liver 2012; 6: 334–8. 22844561 10.5009/gnl.2012.6.3.334 PMC3404170

[4] Strate LL , Liu YL , Huang ES , Giovannucci EL , Chan AT . Use of aspirin or nonsteroidal anti‐inflammatory drugs increases risk for diverticulitis and diverticular bleeding. Gastroenterology 2011; 140: 1427–33. 21320500 10.1053/j.gastro.2011.02.004 PMC3081980

[5] Yamada A , Sugimoto T , Kondo S , *et al*. Assessment of the risk factor for colonic diverticular hemorrhage. Dis Colon Rectum 2008; 51: 116–20. 18085336 10.1007/s10350-007-9137-8

[6] Taki M , Oshima T , Tozawa K , *et al*. Analysis of risk factor for colonic diverticular bleeding and recurrence. Medicine (Baltimore). 2017; 96: e8090. 28930849 10.1097/MD.0000000000008090 PMC5617716

[7] Yuhara H , Corley DA , Nakahara F , *et al*. Aspirin and non‐aspirin NSAIDs increase risk of colonic diverticular bleeding: A systematic review and meta‐analysis. J Gastroenterol 2014; 49: 992–1000. 24221694 10.1007/s00535-013-0905-z

[8] Nagata N , Niikura R , Aoki T , *et al*. Colonic diverticular hemorrhage associated with the use of nonsteroidal anti‐inflammatory drugs, low‐dose aspirin, antiplatelet drugs, and dual therapy. J Gastroenterol Hepatol 2014; 29: 1786–93. 24720424 10.1111/jgh.12595

[9] Chan FKL , Leung Ki En‐L , Wong GLH , *et al*. Risk of bleeding recurrence and cardiovascular events with continued aspirin use after lower gastrointestinal hemorrhage. Gastroenterology 2016; 151: 271–7. 27130815 10.1053/j.gastro.2016.04.013

[10] Sengupta N , Feuerstein JD , Patwardhan VR , *et al*. The risk of thromboembolism vs. recurrent gastrointestinal bleeding after interruption of systemic anticoagulation in hospitalized inpatients with gastrointestinal bleeding: A prospective study. Am J Gastroenterol 2015; 110: 328–35. 25512338 10.1038/ajg.2014.398

[11] Chai‐Adisaksopha C , Hillis C , Monreal M , Witt D , Crowther M . Thromboembolic events, recurrent bleeding and mortality after resuming anticoagulant following gastrointestinal bleeding. A meta‐analysis. Thromb Haemost 2015; 114: 819–25. 26018236 10.1160/TH15-01-0063

[12] Ruff CT , Giugliano RP , Braunwald E , *et al*. Comparison of the efficacy and safety of new oral anticoagulants with warfarin in patients with atrial fibrillation: A meta‐analysis of randomized trials. Lancet 2014; 383: 955–62. 24315724 10.1016/S0140-6736(13)62343-0

[13] Caldeira D , Barra M , Ferreira A , *et al*. Systematic review with meta‐analysis: The risk of major gastrointestinal bleeding with non‐vitamin K antagonist oral anticoagulants. Aliment Pharmacol Ther 2015; 42: 1239–49. 26434935 10.1111/apt.13412

[14] Wang K‐L , Lip GYH , Lin S‐J , Chiang C‐E . Non‐vitamin K antagonist oral anticoagulants for stroke prevention in Asian patients with nonvascular atrial fibrillation: Meta‐analysis. Stroke 2015; 46: 2555–61. 26304863 10.1161/STROKEAHA.115.009947 PMC4542566

[15] Vajravelu RK , Mamtani R , Scott FI , Waxman A , Lewis JD . Incidence, risk factors, and clinical effects of recurrent diverticular hemorrhage: A large cohort study. Gastroenterology 2018; 155: 1416–27. 30056095 10.1053/j.gastro.2018.07.026 PMC6219900

[16] Turpin M , Gregory P . Direct oral anticoagulant use and risk of diverticular hemorrhage: A systematic review of the literature. Can J Gastroenterol Hepatol 2019; 2019: 1. 10.1155/2019/9851307 PMC6604480 31316948

[17] Oakland K , Desborough MJ , Murphy MF , Schachter M , Jairath V . Rebleeding and mortality after lower gastrointestinal bleeding in patients taking antiplatelets or anticoagulants. Clin Gastroenterol Hepatol 2019; 17: 1276–84.e3. 29277620 10.1016/j.cgh.2017.12.032

[18] Strate LL , Gralnek IM . ACG clinical guideline: Management of patients with acute lower gastrointestinal bleeding. Am J Gastroenterol 2016; 111: 459–74. 26925883 10.1038/ajg.2016.41 PMC5099081

[19] Oakland K , Chadwick G , East JE , *et al*. Diagnosis and management of acute lower gastrointestinal bleeding: Guideline from the British Society of Gastroenterology. Gut 2019; 68: 776–89. 30792244 10.1136/gutjnl-2018-317807

[20] Okamoto T , Watabe H , Yamada A , Hirata Y , Yoshida H , Koike K . The association between arteriosclerosis related diseases and diverticular bleeding. Int J Colorectal Dis 2012; 27: 1161–6. 22584295 10.1007/s00384-012-1491-x

[21] Niikura R , Nagata N , Yamada A , Akiyama J , Shimbo T , Uemura N . Recurrence of colonic diverticular bleeding and associated risk factors. Colorectal Dis 2012; 14: 302–5. 21692963 10.1111/j.1463-1318.2011.02611.x

[22] Nishikawa H , Maruo T , Tsumura T , Sekikawa A , Kanesaka T , Osaki Y . Risk factors associated with recurrent hemorrhage after the initial improvement of colonic diverticular bleeding. Acta Gastroenterol Belg 2013; 76: 20–4. 23650778

[23] Fujino Y , Inoue Y , Onodera M , *et al*. Risk factors for early re‐bleeding and associated hospitalization in patients with colonic diverticular bleeding. Colorectal Dis 2013; 15: 982–6. 23560619 10.1111/codi.12232

[24] Kitagawa T , Katayama Y , Kobori I , Fujimoto Yo , Tamano M . Predictors of 90‐day colonic diverticular recurrent bleeding and readmission. Intern Med 2019; 58: 2277–82. 31118377 10.2169/internalmedicine.2276-18 PMC6746631

